# Factors behind Leisure-Time Physical Activity Behavior Based on Finnish Twin Studies: The Role of Genetic and Environmental Influences and the Role of Motives

**DOI:** 10.1155/2014/931820

**Published:** 2014-04-08

**Authors:** Sari Aaltonen, Urho M. Kujala, Jaakko Kaprio

**Affiliations:** ^1^Department of Public Health, The Hjelt Institute, University of Helsinki, P.O. Box 41, Mannerheimintie 172, 00014 Helsinki, Finland; ^2^Department of Health Sciences, University of Jyväskylä P.O. Box 35, 40014 Jyväskylä, Finland; ^3^Institute for Molecular Medicine, University of Helsinki, P.O. Box 21, Tukholmankatu 8, 00014 Helsinki, Finland; ^4^Department of Mental Health and Substance Abuse Services, National Institute for Health and Welfare, P.O. Box 30, 00271 Helsinki, Finland

## Abstract

Different approaches are being taken to clarify the role of various factors in the development of physical activity behaviors. Genetic studies are a new area of physical activity research and also the motives for physical activity have been widely studied. The purpose of this paper is to review the findings emerging from the longitudinal genetic studies on leisure-time physical activity and to evaluate the associations between motivational factors and leisure-time physical activity. The focus is to review recent findings of longitudinal Finnish twin studies. The results of the latest longitudinal Finnish twin studies point to the existence of age-specific genetic and environmental influences on leisure-time physical activity. Variations in environmental factors seem to explain the observed deterioration in leisure-time physical activity levels. A decline in genetic influences is seen first from adolescence to young adulthood and again from the age of thirty to the mid-thirties. In the Finnish twin participants, mastery, physical fitness, and psychological state were the major motivation factors associated with consistent leisure-time physical activity behavior. The results also indicate that intrinsic motivation factors may be important for engagement in leisure-time physical activity.

## 1. Introduction

Epidemiological studies have revealed that physical activity can reduce risks for obesity as well as preventing several chronic diseases and even reducing mortality [1–6]. However, a substantial proportion of individuals, especially those living in the most developed countries, do not participate in sufficient physical activities and thus fail to gain the subsequent health benefits [7, 8]. If we are to understand why some subjects fail to engage in regular physical activity in leisure time, then we need to clarify which factors underlie individual differences in physical activity behavior.

It is known that many different factors play a role in leisure-time physical activity behavior. Leisure-time physical activity level may partly be determined on the basis of personal traits, needs, and interests and partly on external factors such as environment and availability factors [9–11]. Some of these factors may make it easier or harder for certain individuals to achieve high levels of physical activity. However, it is important to remember that environmental and genetic factors always work in conjunction. In the last decades, serious attempts have been made to clarify the role of different factors in physical activity behavior. Studies have concentrated on the correlates (i.e., factors associated with physical activity) and the determinants of physical activity (i.e., factors associated with a causal relationship). No clear consensus has been achieved, although several factors such as age, sex, previous physical activity, self-efficacy, and health status do seem to be associated with current physical activity level [11].

Genetic studies are one of the new areas of physical activity research. This is logical because individual's genetic characteristics seem to be a possible determinant of physical activity [11] and advances in genetic technologies permit identification of individual genes or gene systems associated with a trait such as physical activity. These studies have attempted to determine the genetic architecture of factors contributing to an individual's propensity to be physically active. This includes estimating the overall role of genetic factors (in contrast to all nongenetic factors). If genetic factors are shown to be relevant, work is done to identify the genes and the mode of action of the genes in physical activity. The overall contribution of genetic factors to variation in physical activity is often examined by conducting twin studies. Twin study designs are popular in behavioral genetics, as they provide an opportunity to disentangle the effects of genes from those of the environment [12, 13]. In addition to genetics, motivation is a personal characteristic that also may be one of the key factors to help understand why some people spend their leisure time undertaking physical activity. This may be the reason why motives have been widely studied.

Although there are cross-sectional studies examining the associations between the genetic and environmental influences, motives, and leisure-time physical activity, longitudinal studies have been less frequently conducted. However, the advantages of longitudinal study designs are that causal associations can be better revealed and that the true effects of aging may be demonstrated. To date, little is also known about whether the motives for physical activity change over the life course. Another poorly characterized area is the difference in motivational factors between active and inactive individuals. The Finnish twin cohorts offered a great opportunity to utilize longitudinal study design and conduct comparison between physically active and inactive twins. The main aim of the present paper is to review the recent findings on genetic and environmental influences on the longitudinal changes of leisure-time physical activity behavior as revealed in the Finnish twin studies: first, from adolescence to young adulthood and, second, over a 6-year follow-up period in adulthood. Furthermore, the motives for leisure-time physical activity among consistently physically active and inactive people from the Finnish twin studies are presented. The present paper is based on the Ph.D. thesis of the first author, Aaltonen [14].

Physical activity has been defined to be body movements produced by the skeletal muscles, which cause a substantial increase in energy demands over resting energy expenditure [15]. However, the term physical activity is often used interchangeably with the terms exercise or sports although that is not correct or recommended [15]. The choice of term (physical activity, exercise, or sports) may impact the results of the genetic analyses and motivational studies. In this review, we have therefore used the same terms used in the original papers.

## 2. Genetic and Environmental Influences on Leisure-Time Physical Activity

In quantitative genetic modeling, physical activity is assumed to be made up of genetic and environmental contributions. Environmental influences can be divided into shared environmental influences, representing the effects of environmental factors shared, for example, by the cotwins in a pair. Specific environmental influences represent unique environmental influences and specific environmental influences result in differences between the cotwins of a pair [16]. A number of twin studies using the quantitative genetic modeling have shown that genetic influences play an important role in explaining individual differences in leisure-time physical activity [17–26]. However, the different studies have found very different patterns. The largest of these studies has pooled data on leisure-time exercise behavior from seven different countries (GenomEUtwin project) and found that the heritability of exercise participation ranged from 48% to 71%, with the exception of Norwegian men where it was only 27% [22]. As this investigation indicates, it is clear that there is heterogeneity in the results of studies related to genetic influences on leisure-time physical activity. It can be assumed that a significant proportion of the heterogeneity may derive not only from changes in the genetic contribution to this trait in different aged individuals but also from culture-specific, sex-specific, and period-specific effects. Physical activity assessment methods may also have an influence on the heterogeneity of results. Heritability is always assessed at a particular time and age, and above all, heritability is an estimate of the genetic influences to individual differences on a population level.

Longitudinal study designs are needed to reveal the age-specific genetic influences on leisure-time physical activity. However, only a few studies have investigated the genetic and environmental influences on longitudinal leisure-time physical activity before the Finnish twin studies were published [27, 28]. Simonen et al. [29] reported change across the lifespan in heritability estimates for leisure-time physical activity in adult male twin pairs. A recent comparative study in twins aged 19 to 50 from seven countries that collaborated in the GenomEUtwin project was not a pure longitudinal study, but it revealed also age-related changes in heritability [30].

Earlier studies have also reported a shift between genetic and environmental influences in the time periods between childhood and adolescence and between adolescence and young adulthood, although at different times in different studies and in different directions. In Dutch boys, genetic influences on leisure-time exercise behavior were fluctuating from age of 7 years to age of 12 years, while in girls genetic influences were more stable [31]. In this study, shared environmental influences mainly explained the largest part of the variance in leisure-time exercise behavior between childhood and early adolescence. The decline in the heritability estimate was noted in longitudinal studies by both van der Aa et al. [32] and Eriksson et al. [33]. Genetic influences on leisure-time physical activity declined from early adolescence to late adolescence in both sexes in Dutch twins [32] and decline was also seen during a 4-year followup among young Swedish men in their twenties [33]. In contrast to these studies, Stubbe et al. [34] found in their longitudinal study that between the age of 13 and the age of 16 years genetic influences were not important, whereas between the age of 19 and the age of 20 years genetic influences largely explained the individual differences in leisure-time sports participation.

### 2.1. Genetic and Environmental Influences on Longitudinal Leisure-Time Physical Activity in Finnish Twin Studies

**Figure 1:**
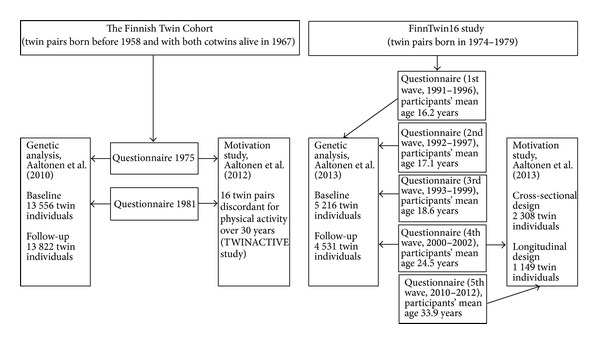
Participants in the Finnish twin studies originally described by Aaltonen et al. [27, 28, 65, 66].

The participants of the Finnish twin studies examined for genetic and environmental influences of leisure-time physical activity are drawn from two cohorts: the FinnTwin16 study (twins born between 1975 and 1979) and the older Finnish Twin Cohort (twins born before 1958 and both cotwins alive in 1967) (Figure 1). Both cohorts were identified from the Central Population Registry of Finland with the purpose of forming a national resource for genetic epidemiological studies [35–39]. The longitudinal quantitative genetic analyses of these cohorts published by Aaltonen et al. [27, 28] produced results, which corroborate the findings of much of the previous work in this field; that is, the heritability of leisure-time physical activity behavior ranged between 27% and 71% as summarized above. In the studies by Aaltonen et al. [27, 28], the heritability of leisure-time physical activity ranged between ∼30% and ∼52%.

**Figure 2:**
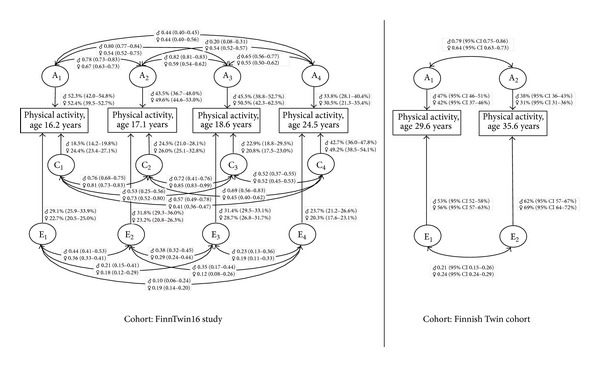
The summary of the final genetic models for leisure-time physical activity between both ages of 16.2 and 24.5 years and ages of 29.6 and 35.6 years in Finnish twin studies. It is important to note that the cohorts used in the models between ages of 16.2 and 24.5 years and between ages of 29.6 and 35.6 years are not identical. Genetic and environmental influences are shown as percentages; upper value is for men and lower value is for women. Confidence intervals (95% CI) are shown in the parentheses. Additive genetic, shared environmental, and specific environmental correlations between the baseline and follow-up results are shown as curved arrows. The more detailed summaries for models are presented in the publications of Aaltonen et al. [27, 28].

In addition, these results of the Finnish twin studies confirmed the existence of age-specific changes in the genetic and environmental influences on leisure-time physical activity. The results revealed a change in the pattern of genetic and environmental influences in the progress of leisure-time physical activity: first, from adolescence to adulthood [27] and, second, from the age of thirty to the mid-thirties [28]. The summary of the final models for leisure-time physical activity has been presented in Figure 2.

In the study of the younger Finnish twins, the relative role of additive genetic influences remained rather stable during adolescence only changing from 43% to 52% [27]. However, the heritability estimate declined in the period from adolescence to young adulthood to around 30%. This decrease in genetic influences is parallel to the indications that leisure-time physical activity level declines with age [8, 40–42]. Shared environmental influences, in turn, also showed relative stability during adolescence, but in contrast to genetic influences they increased markedly in young adulthood, especially in women. Additive genetic, shared environmental, and specific environmental correlations between the baseline results in adolescence and follow-up results in young adulthood are shown in Figure 2.

In adulthood, around the age of thirty, additive genetic influences were also moderate, at 44%, while a slight decline was also seen in the mid-thirties, when additive genetic influences were estimated to be 34% [28]. In this study, the additive genetic correlation for leisure-time physical activity was greater for men, 0.79, than for women, 0.64, but the environmental correlation between the two time points did not differ substantially between the sexes (Figure 2). The longitudinal phenotypic correlation in men was 0.45, of which 74% was due to longitudinal additive genetic influences, while in women the longitudinal phenotypic correlation was 0.38, of which 60% was due to longitudinal additive genetic influences.

Based on these longitudinal quantitative studies among Finnish twins, both shared and specific environmental influences affected leisure-time physical activity up to adulthood, but only specific environmental influences were further present in adulthood in the thirties and mid-thirties. In contrast to the consistent expression of an important group of genes observed in adulthood, new additive genetic, shared, and specific environmental influences emerged at each follow-up point in adolescence and in young adulthood.

## 3. Motives for Leisure-Time Physical Activity

In addition to genetics, motivation is a personal characteristic that also may be one of the key factors for understanding why some people are physically active in their leisure time. Many studies have been published on what motivates individuals to undertake physical activity. Several of these studies have reported that, regardless of age, gender, or level of physical activity, health is an important factor motivating participation in leisure-time physical activity among adults [43–49]. For instance, among the citizens of the European Union member states, almost half of those aged over 15 years reported good health as the most important reason for participation in physical activity [44]. Despite the general importance of health as a factor motivating leisure-time physical activity, it seems to be a factor which varies by region [50]. In addition to health benefits, appearance [51], fitness [48], enjoyment [48], and body image [52] are features which are highly linked to physical activity among young adults. However, it is important to remember that motives may change during the stages of adoption of some form of physical exercise [53]. Differences may also exist according to exercise type [54, 55], gender, and age [46, 53, 56, 57].

So far, only some of the published studies have examined differences in motivational factors between physically active and inactive people, but none of these studies has been longitudinal. Studies have been based on the hypothesis that the level of leisure-time physical activity is explained by differences in motivational factors. One study did indicate that physical activity was mostly associated with environmental factors, whereas inactivity was linked with sociodemographic factors [58]. Overall, when physically active people were compared to physically inactive people, health, fitness, and enjoyment were identified as the major motivational factors for leisure-time physical activity in the active people [46, 48, 59]. Social reasons were highlighted by physically active and inactive people in the recent study of Costello et al. [60]. In this study, physically inactive people wanted leisure-time physical activity to be purposeful and fun, while the active participants enjoyed exercise regardless of its purpose. The randomized controlled study of Silva et al. [61] found that women whose intervention focused on promoting autonomous forms of exercise regulation and intrinsic motivation showed higher physical activity levels than controls.

The role of family and genetic factors in motivation for physical activity is poorly studied; further, links between physical activity, genetic influences, and motivational factors remain unraveled. A recent animal study suggested that voluntary running motivation may be inherent [62]. In a study by Huppertz et al. [63], exercise attitude components explained 28% of the variance in leisure-time exercise behavior. In bivariate modeling, all the genetic and all but two unique environmental correlations between attitude components and exercise behavior suggested a causal relationship between exercise attitude and leisure-time exercise behavior. The authors concluded that both exercise attitudes and exercise behavior are heritable and are partly correlated through pleiotropic genetic effects. It thus seems plausible that family and genetic factors influence motives for physical activity.

### 3.1. Motives for Leisure-Time Physical Activity Based on Finnish Twin Studies

The motives for undertaking leisure-time physical activity were also studied using data from the FinnTwin16 study (younger twins born between 1975 and 1979) and the Finnish Twin Cohort (older twins born before 1958 and both cotwins alive in 1967) (Figure 1). Participants from the FinnTwin16 study were analyzed as individuals in their mid-thirties. The cotwin control study design was used when twin pairs (mean age 60.4 years) discordant for leisure-time physical activity over 30 years from the Finnish Twin Cohort were analysed. In these studies, physical fitness, psychological state, and enjoyment were the highest scored reasons for engaging in leisure-time physical activity when motivational factors were assessed by the Recreational Exercise Motivation Measure (REMM) [64]. Thus, the same factors seem to be important for engagement in leisure-time physical activity among both younger and older adults in Finland. These were also the factors that the physically active participants rated higher than the physically inactive participants. The findings of the importance of physical and psychological health as motivational factors are also in agreement with earlier findings by other researchers presented above.

**Figure 3:**
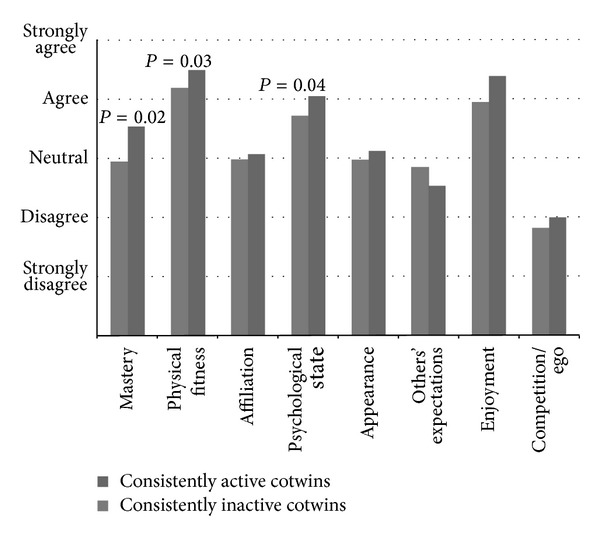
Differences in the subdimensions of the REMM measurement scale among twin pairs who have been discordant for leisure-time physical activity over 30 years (twins from the Finnish Twin Cohort) [65]. The dark grey columns of the histogram represent twins who have been physically active over 30 years and the light grey columns represent their inactive cotwins. The names of the subdimensions are shown below the columns and the answer options are shown on the left hand side of the histogram. The *P* values above the columns indicate that there is a statistical difference between the active and inactive cotwins. The *P* value is shown only if a significant difference between the groups was detected.

**Figure 4:**
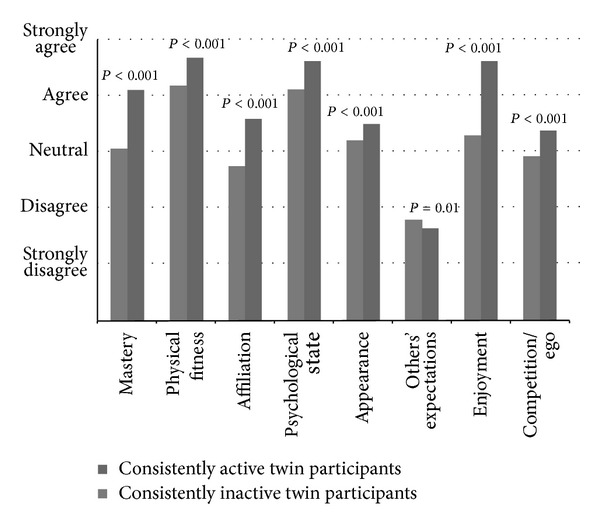
Differences in the subdimensions of the REMM measurement scale among consistently physically active and inactive twin individuals in their mid-thirties (twins from the FinnTwin16 study) [66]. The dark grey columns of the histogram represent twin individuals who have been physically active for at least the last ten years and the light grey columns represent twin individuals who have been inactive for the same period of time. The names of the subdimensions are shown below the columns and the answer options are shown on the left hand side of the histogram. The *P* values above columns indicate that there is a statistical difference between the active and inactive cotwins.

However, a major result of the Finnish twin studies related to motives confirmed the importance of motivational factors in separating leisure-time physical activity behavior. When motives for leisure-time physical activity were measured among older Finnish twin pairs who have been discordant for leisure-time physical activity over 30 years, the motivational factors of mastery, physical fitness, and psychological state were subdimensions that differed significantly between the consistently physically active twins and their consistently physically inactive cotwins [65] (Figure 3). The same results were obtained when the consistently active twin individuals were compared to the consistently inactive twin individuals in the FinnTwin16 study [66] (Figure 4). These younger twin individuals had been either consistently physically active or consistently physically inactive for at least the last ten years. Moreover, motivational factors related to appearance, enjoyment, and willingness to be fitter or look better than others and the social aspect of physical activity differed also significantly between the younger twin individuals in the FinnTwin16 study [66] (Figure 4). The results did not substantially differ according to gender.

In the Finnish twin studies, both younger twin individuals and older twin pairs rated conforming to others' expectations as the least meaningful motivating factors for undertaking leisure-time physical activity. Conforming to others' expectations is one of the subdimensions of the REMM. The older inactive twins in the Finnish Twin Cohort emphasized compliance with other peoples expectations slightly more than their active cotwins within the pair. The same result was found among younger twin individuals in the FinnTwin16 study. However, the difference was statistically significant only between the consistently active and consistently inactive twin individuals in the FinnTwin16 study and between the consistently active and consistently inactive women in their mid-thirties in the FinnTwin16 study [66]. No statistically significant difference was seen between the consistently active and consistently inactive men in their mid-thirties in the FinnTwin16 study [66] or between the twin pairs who have been discordant for leisure-time physical activity over 30 years in the Finnish Twin Cohort [65]. The measure of effect size also revealed that the difference between the groups was of low magnitude. The subdimension of conforming to others' expectations clearly reflects the extrinsic type of motivation. This suggests that genetic factors may contribute to the relationship of physical activity and motivations, but this has not been formally modelled.

## 4. Conclusions

In conclusion, several studies have provided evidence that both genetic and environmental influences and motives are associated with physical activity behavior. Furthermore, the latest longitudinal studies among Finnish twins deepened the understanding of regular, consistent leisure-time physical activity behavior. Specifically, the results of the longitudinal Finnish twin studies found evidence for the existence of age-specific genetic and environmental influences on leisure-time physical activity. Such age-specific genetic effects need to be carefully considered when designing and analyzing molecular genetic studies to identify specific genes and factors affecting the expression of genes, such as through epigenetic mechanisms. In addition, the results of the Finnish twin studies revealed differences in motivational factors influencing leisure-time physical activity between consistently physically active and inactive people. The results also indicated that intrinsic motivation factors are important for engagement in leisure-time physical activity.

The results of the present review suggested that variations in environmental factors seemed to explain the observed deterioration in leisure-time physical activity levels. Measures promoting leisure-time physical activity may be even more important for women than for men, because of the greater role of environmental influences in women shown by these Finnish twin studies. Furthermore, the transitional period from adolescence to young adulthood should be seen as a strategic point to stimulate leisure-time physical activity that would also lead to an active lifestyle in later adulthood.
